# Supplementary material for: Sandbur Drought Tolerance Reflects Phenotypic Plasticity Based on the Accumulation of Sugars, Lipids, and Flavonoid Intermediates and the Scavenging of Reactive Oxygen Species in the Root
Source: Int J Mol Sci. 2021 Nov 23;22(23):12615. doi: 10.3390/ijms222312615 (PMC8657935; doi:10.3390/ijms222312615)
Supplement: Supplementary file 1 [file ijms-22-12615-s001.zip › Supplementary figures and tables.pdf]

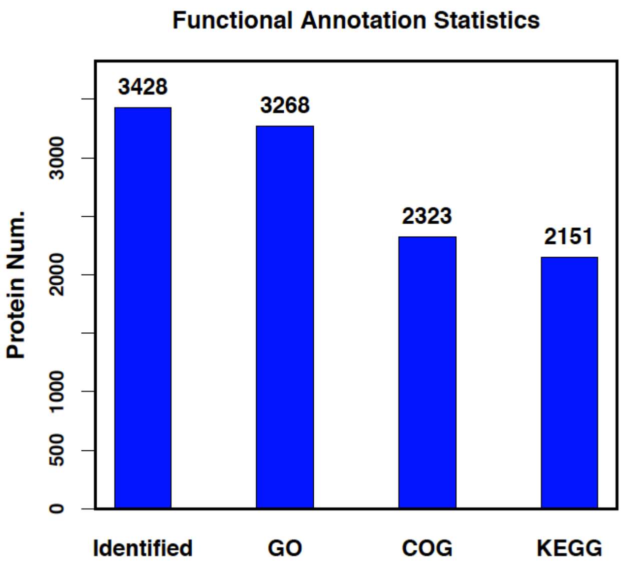

Figure S1. Functional annotation of proteins differing in abundance between *C. spinifex* roots under normal and drought stress conditions as revealed in the iTRAQ dataset based on GO categories, COGs and KEGG pathways.

Table S2. Unique primers for nine candidate genes amplified by real-time PCR.

Gene Specific Primer Name	Primer
Rp S3a-F	TCTTCAGAGTGATGAAGATC
Rp S4-F	ATCTTCTGGTCCTCACAAGT
Rp S7-F	TCAGATACCGTTTGGATGGT
Rp S15a-1-F	ATGGTGAGAGTCAGCGTCCT
Rp S19-F	AGATGGTTGGCCACTACCTC
Rp L4-1-F	CGAGAGTGCCTTCAAGAAGC
Rp L10-F	ACCCATACCGTAGGTGAAGG
Initiation factor-F	ATTAACCAGGGAGACATCAT
ABC transporter-F	ATACAAGGCAGCTGTTGAGG
Actin-F	TTCCATTGTCACATACAAG
Rp S3a-R	AGTGGTCTTCACATCAACAT

Rp S4-R	GTGTCATACAGAAGCCTGTA
Rp S7-R	AGACCTTCACTGCAGTCTAC
Rp S15a-1-R	CTCCCATTCAACTCAACCAC
Rp S19-R	TTCATGCGTCACCAAGTCAT
Rp L4-1-R	TCATACCGGGCTAAGCTTGG
Rp L10-R	ATGTCCCAGCTGGAAACA
Initiation factor-R	GCAAATCGGCTTGACTAGAT
ABC transporter-R	GTTGCATTACCTAACGACAT
Actin-R	AATCTGTAGCAAATGGCTGA
